# Supplementary material for: ATRX, DAXX or MEN1 mutant pancreatic neuroendocrine tumors are a distinct alpha-cell signature subgroup
Source: Nat Commun. 2018 Oct 12;9:4158. doi: 10.1038/s41467-018-06498-2 (PMC6185985; doi:10.1038/s41467-018-06498-2)
Supplement: Supplementary file 1 — Supplementary Information [file 41467_2018_6498_MOESM1_ESM.pdf]

Supplementary Information for Laddha SV and Chan SC et al., :

ATRX, DAXX or *MEN1* mutant pancreatic neuroendocrine tumors are a distinct "alpha-cell signature" subgroup

Supplementary Tables

Supplementary Table 1: IHC staining results of 36 PanNETs samples

Supplementary Table 2: PEEG Set from three Meta-datasets

Supplementary Table 3: GSEA enrichment scores for all PEEG Set

Supplementary Table 4: Bramswig et al., expression for HNF1A in normal alpha and beta cells and motif TFs analysis on alpha specific genes

Supplementary Figures

Supplementary Fig 1: Unsupervised clustering of top variable number of genes (RNAseq)

Supplementary Fig 2: PCA of top variable number of genes (RNAseq)

Supplementary Fig 3: Top 100 variable genes heatmap showing liver and complement specific genes

Supplementary Fig 4: Validation of subtyping in a larger PanNETs set (n=47)

Supplementary Fig 5: PCA of top variable number of CpG sites (450K methylation)

Supplementary Fig 6: IHC for histone methylation level for H3K36me3 and H3K27me3

Supplementary Fig 7: Alpha and Beta cell lineage specific genes expression

Supplementary Fig 8: APOH gene expression and IHC in two subtypes

Supplementary Figure 1

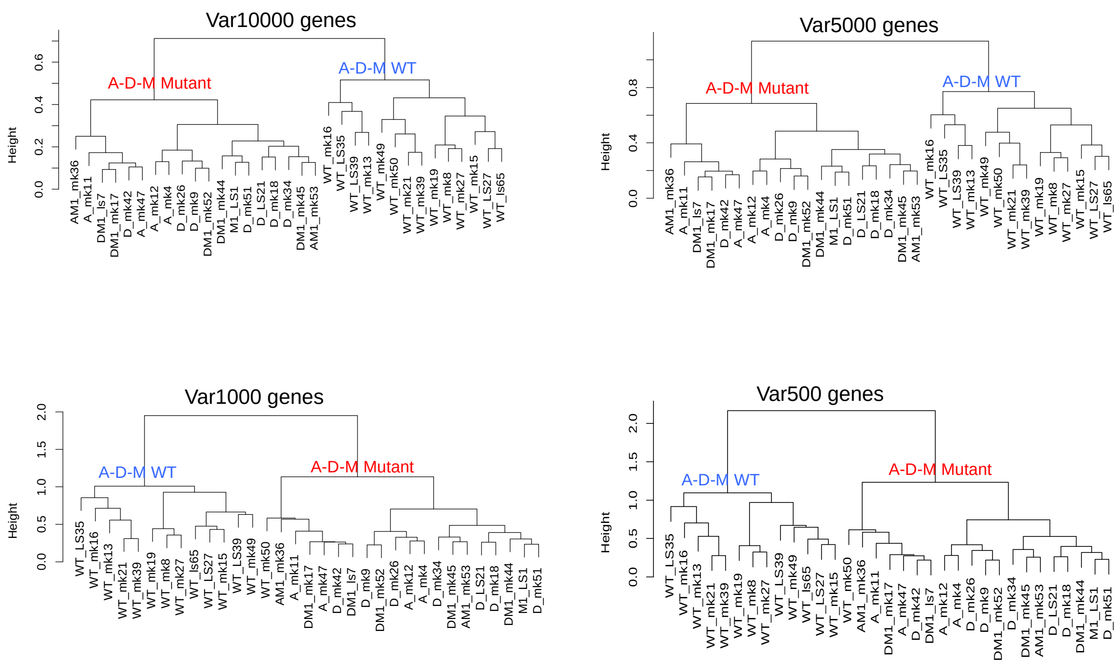

Supplementary Figure 1: Unsupervised clustering of top variable (Var) number of genes for four different gene set (Var10000, Var5000, Var1000 and var500) shows robust subtyping. Var10000 is a gene set with 10000 top variable gene based on gene expression across all samples. All four genes set show robust stratification of A-D-M mutant (in red) from A-D-M WT (in blue) PanNETs.

Supplementary Figure 2

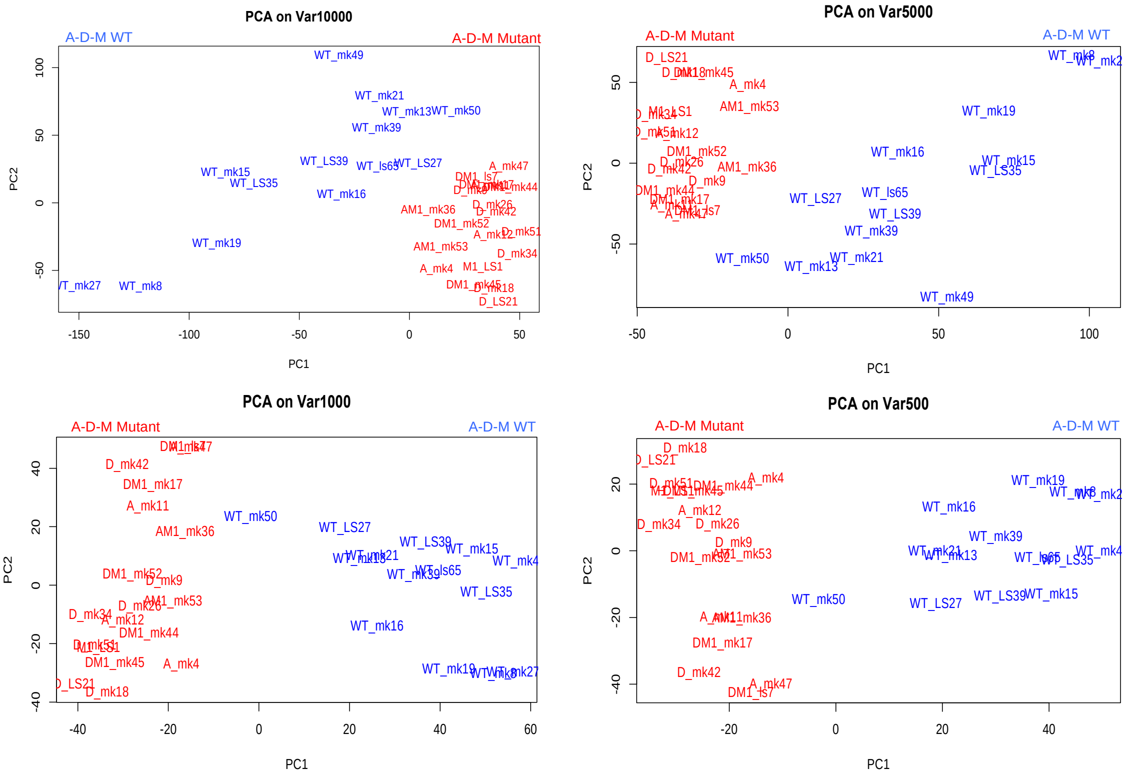

Supplementary Figure 2: Principal Component analysis of top variable (Var) number of genes for four different gene set (Var10000, Var5000, Var1000 and var500) shows robust subtyping. A-D-M mutant are highlighted in red and A-D-M WT panNETs in blue.

Supplementary Figure 3

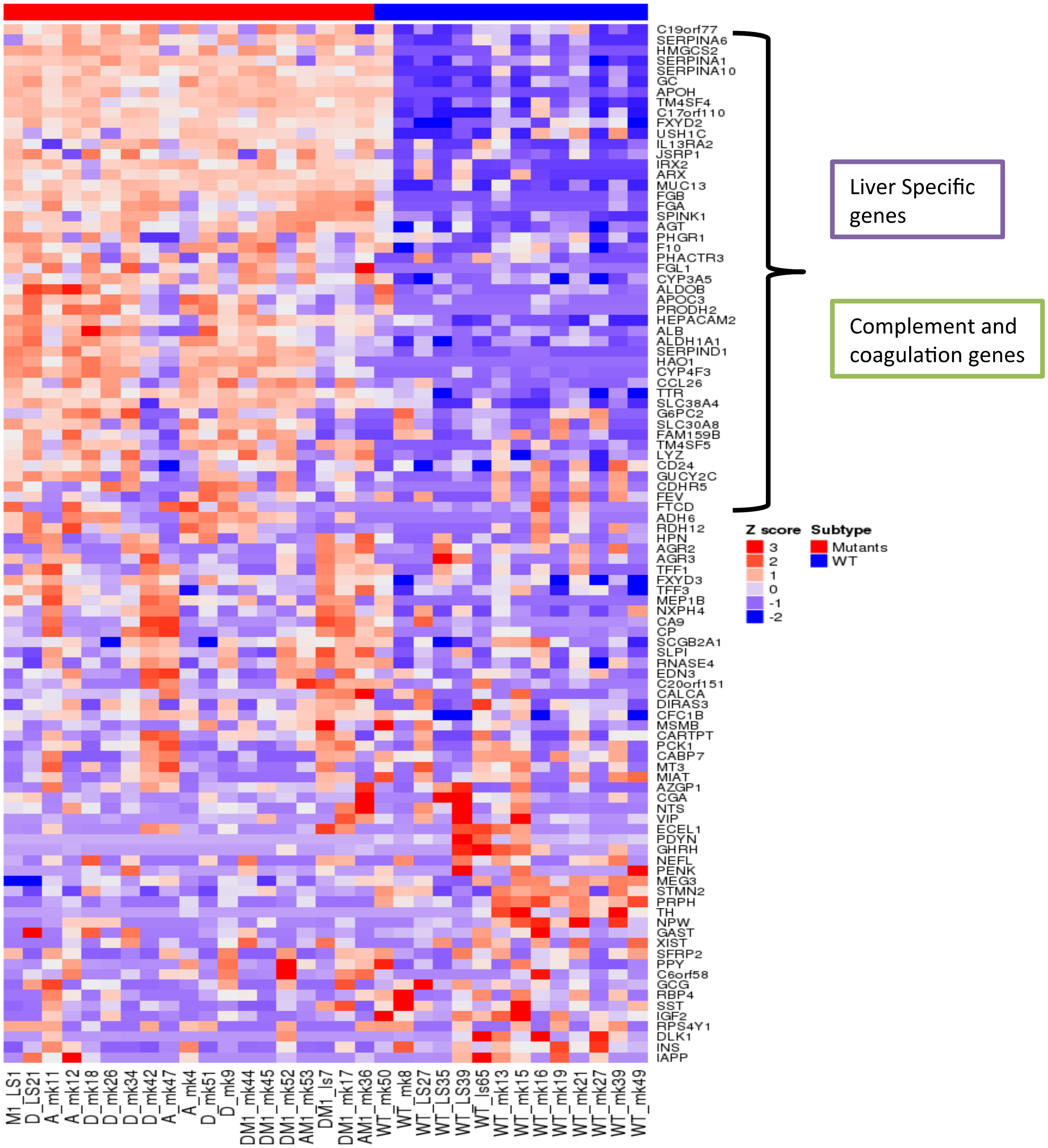

Supplementary Figure 3: Heatmap of top 100 variance genes stratify A-D-M mutant from A-D-M WT panNETs. Many of liver and complement specific genes are highly expressed in A-D-M mutants.

Supplementary Figure 4

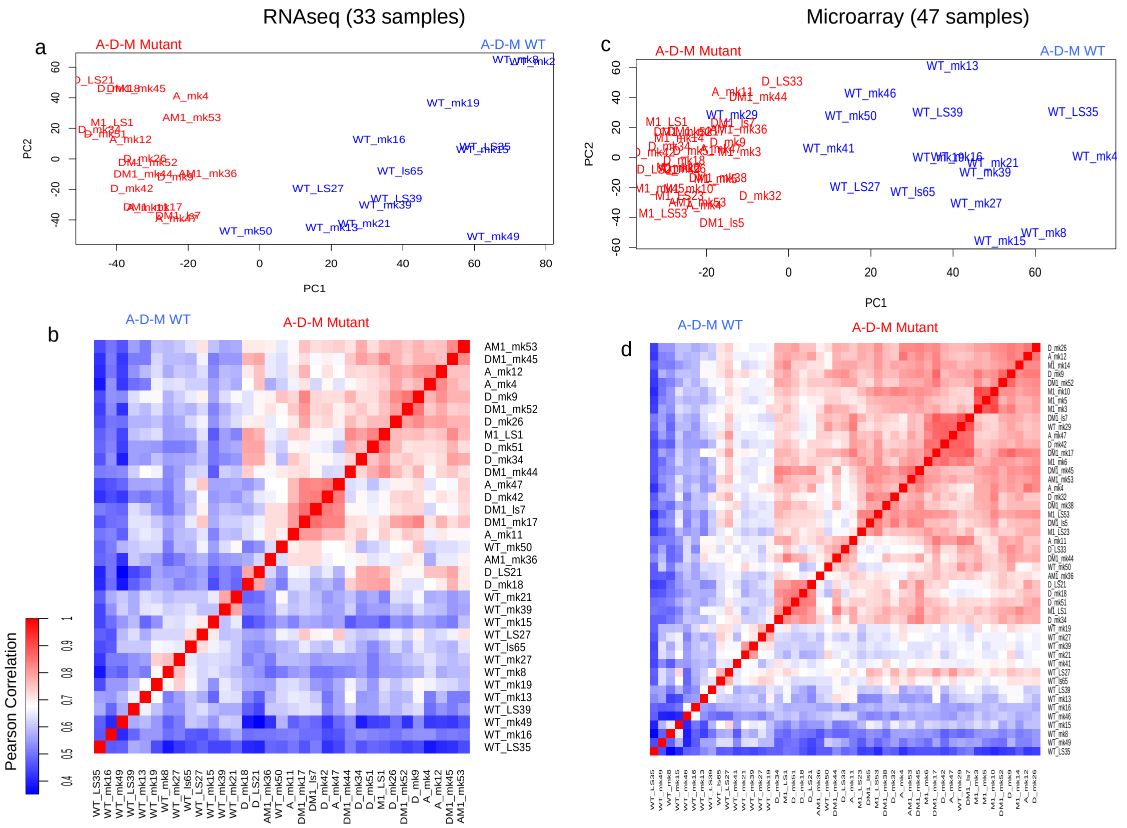

Supplementary Figure 4: Validation of subtyping in a larger PanNETs set (n=47). The distinct gene expression signature and greater degree of homogeneity of A-D-M mutant over A-D-M WT PanNETs were validated in a larger tumor set (47 PanNETs including the 33 PanNETs where RNA sequencing was performed) using gene expression microarray technology (a and b are PCA and Pearson correlation on Var3000 genes from RNAseq and c and d are for Var3000 genes from Microarray.

Supplementary Figure 5

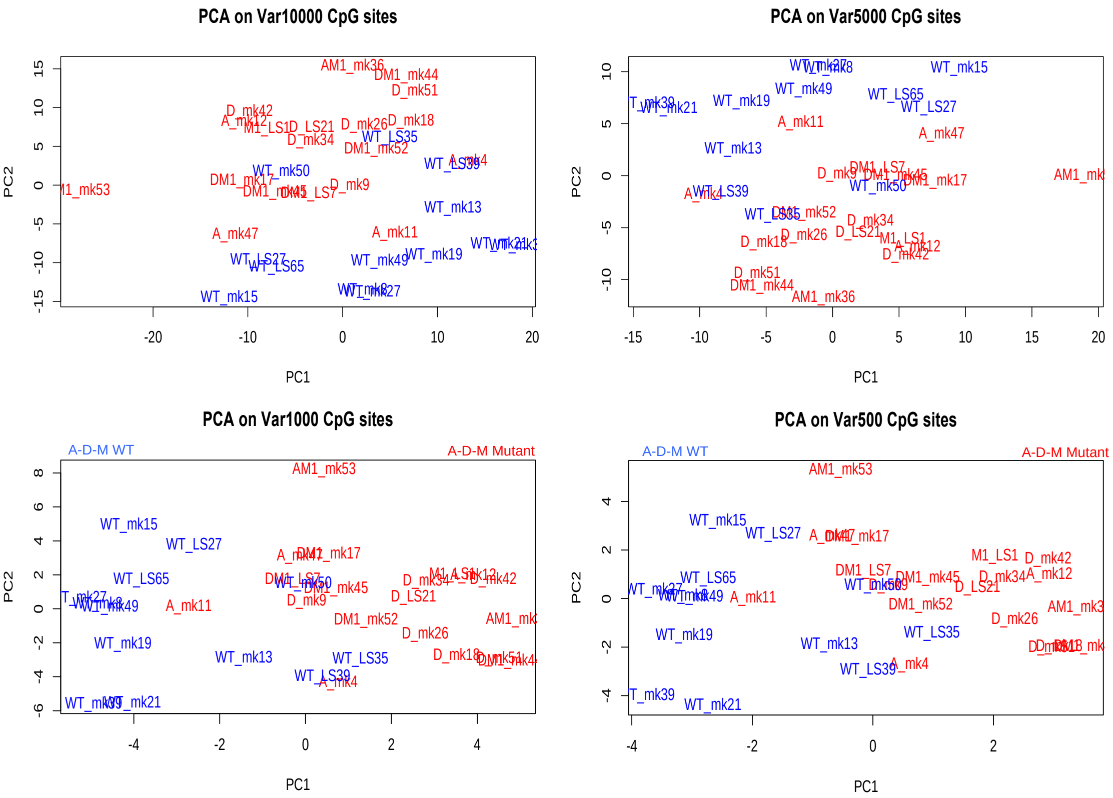

Supplementary Figure 5: PCA of top variable number of CpG sites (450K methylation) for four different gene set (Var10000, Var5000, Var1000 and var500). A-D-M mutant are highlighted in red and A-D-M WT panNETs in blue.

Supplementary Figure 6

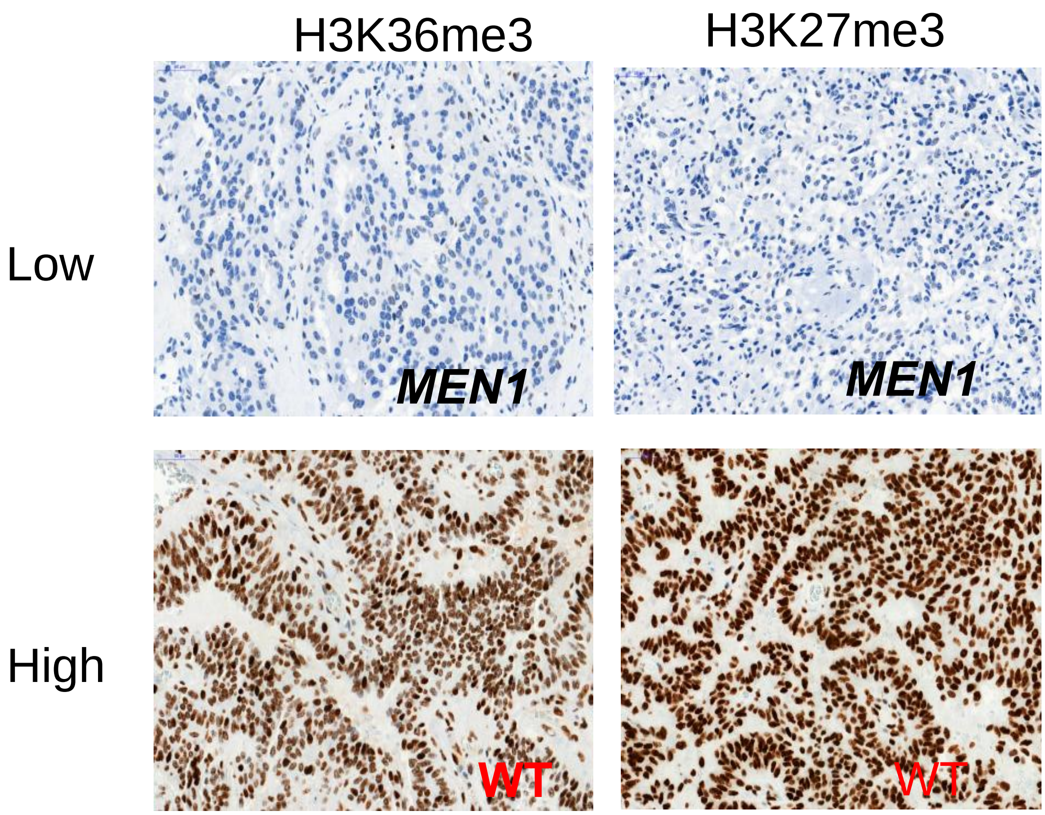

Supplementary Figure 6: Methylation level of H3K36me3 and H3K27me3 in PanNETs. Representative pictures of altered level of histone marks in *MEN1* mutated samples (low) and high in WT PanNETs.

Supplementary Figure 7

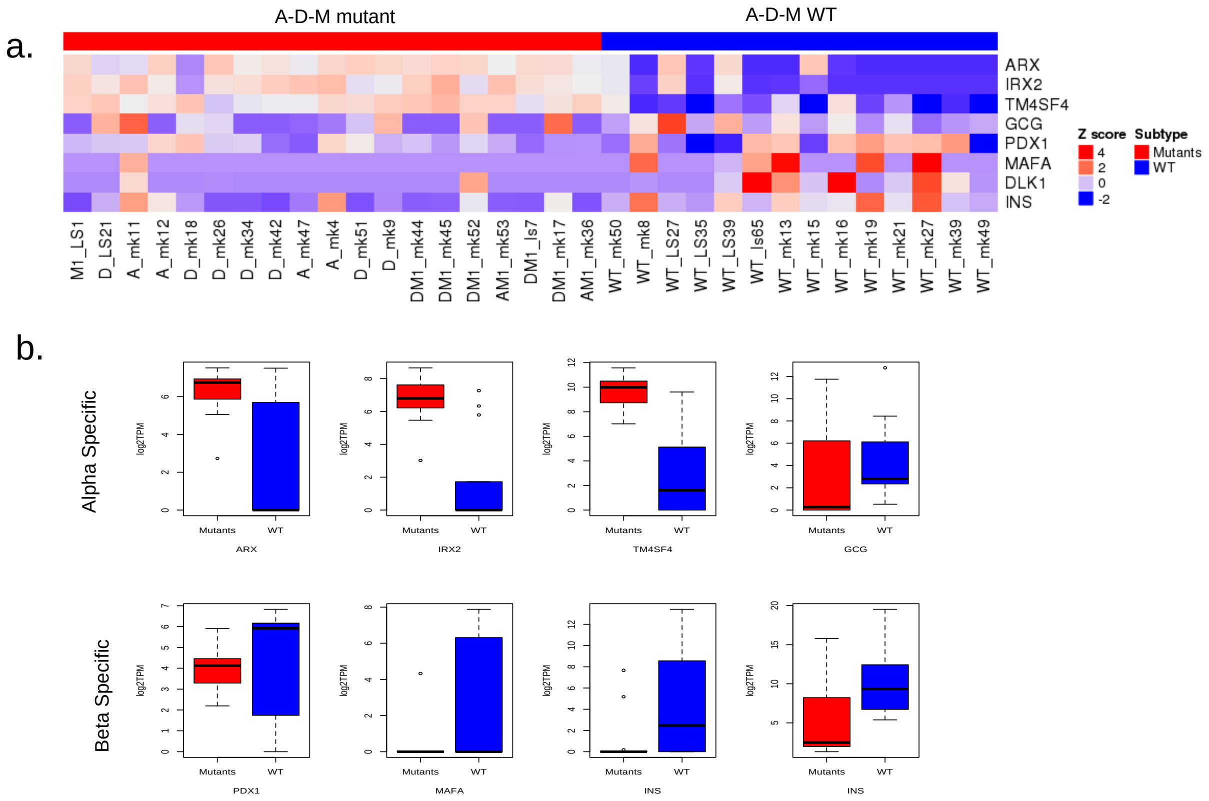

Supplementary Figure 7: Alpha and Beta cell lineage specific genes expression and boxplot for PanNETs subtypes. Alpha Specific genes are *ARX* (TF), *IRX2* (TF), *TM4SF4* (Alpha cell surface marker), GCG and beta Specific genes are *PDX1* (TF), *MAFA* (TF), *INS*, *DLK1*. TFs represent Transcription Factor.

Supplementary Figure 8

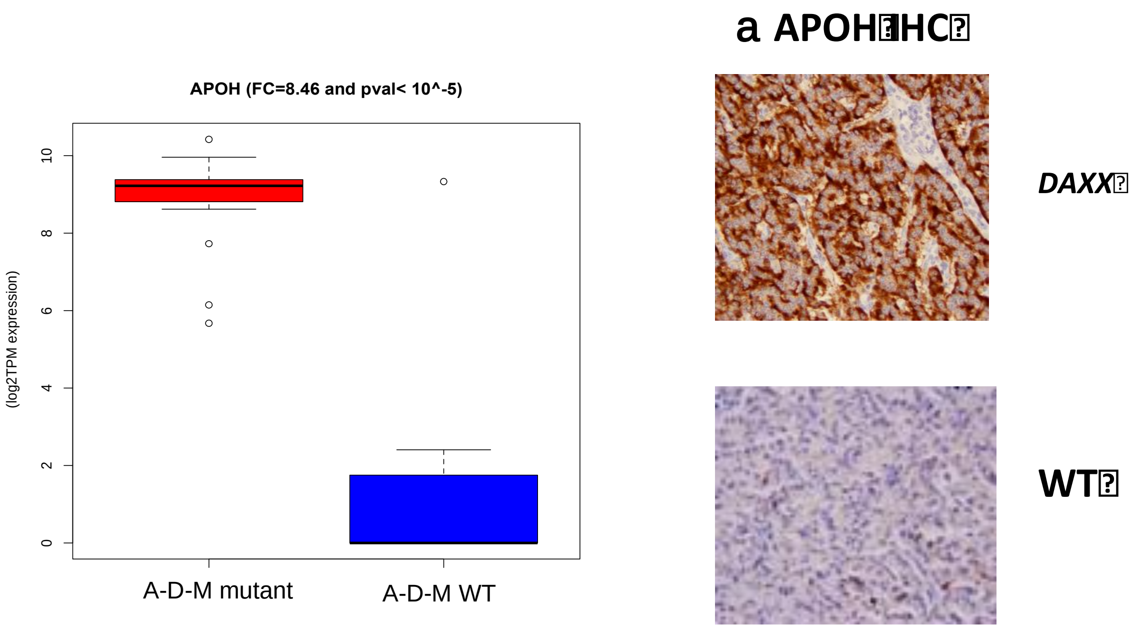

Supplementary Figure 8: APOH gene expression and IHC. A-D-M mutant (red) has higher expression of APOH at mRNA level and Protein level. In consistent with the gene expression profile, APOH protein was strongly expressed in 70±2.5% of mutants and only18±2.0% of WT PanNETs.

Supplementary Tables

Supplementary table 1: Overview of 36 panNETs IHC staining for histone methylation result.

a) Summary table for *MEN1* mutated PanNETs compared with A-D-M WT PanNETs

Mutation	Methylation Level	H3K4me3	H3K9me3	H3K27me3	H3K36me3
MEN1	Low	22%	44%	44%	33%
MEN1 WT	Low	0%	27%	9%	0
	P value	NS	NS	0.05	0.02

*NS = not significant, % represent ration for number of samples with low methylation level divided total number of samples for respective class

b) Overview of histone methylation level for all samples

Mutations	Subtype	H3K4me3	H3K9me3	H3K27me3	H3K36me3
MEN1	A-D-M Mutant	Low	High	Low	High
MEN1	A-D-M Mutant	High	High	High	High
MEN1	A-D-M Mutant	Low	Low	Low	Low
MEN1, ATRX	A-D-M Mutant	High	High	High	High
MEN1, DAXX	A-D-M Mutant	High	High	High	High
MEN1, DAXX	A-D-M Mutant	High	Low	Low	High
MEN1, DAXX	A-D-M Mutant	High	Low	Low	Low
MEN1, DAXX	A-D-M Mutant	High	High	High	Low
MEN1, DAXX	A-D-M Mutant	High	Low	High	High
ATRX	A-D-M Mutant	High	High	High	High
ATRX	A-D-M Mutant	High	High	High	High
DAXX	A-D-M Mutant	High	Low	High	High
DAXX	A-D-M Mutant	High	High	High	High
DAXX	A-D-M Mutant	High	High	High	High
	A-D-M WT	High	High	High	High
	A-D-M WT	High	High	High	High
	A-D-M WT	High	Low	High	High
	A-D-M WT	High	High	High	High
	A-D-M WT	High	Low	High	High
	A-D-M WT	High	Low	Low	High
	A-D-M WT	High	High	High	High
	A-D-M WT	High	High	High	High
	A-D-M WT	High	High	High	High
	A-D-M WT	High	High	High	High
	A-D-M WT	High	High	High	High
	A-D-M WT	High	High	High	High
	A-D-M WT	High	High	High	High
	A-D-M WT	High	High	High	High
	A-D-M WT	High	High	High	High
	A-D-M WT	High	High	High	High
	A-D-M WT	High	Low	High	High
	A-D-M WT	High	High	High	High
	A-D-M WT	High	Low	Low	High
	A-D-M WT	High	Low	High	High
	A-D-M WT	High	High	High	High
	A-D-M WT	High	High	High	High

Supplementary Table 2

Gene Set	PMID	Description	# of Genes in Gene Set
**Wang_adult_alpha.genes**	**27364731**	**Wang_YJ_et_al_2016_Adult_Alpha_Cell.genes**	**212**
**Wang_adult_beta.genes**	**27364731**	**Wang_YJ_et_al_2016_Adult_Beta_Cell.genes**	**376**
**Muraro_Alpha.genes**	**27693023**	**Muraro_MJ_et_al_2016_Alpha_Cell.genes**	**572**
**Muraro_beta.genes**	**27693023**	**Muraro_MJ_et_al_2016_Beta_Cell.genes**	**953**
**Muraro_Delta.genes**	**27693023**	**Muraro_MJ_et_al_2016_Delta_Cell.genes**	**250**
**Muraro_PP.genes**	**27693023**	**Muraro_MJ_et_al_2016_PP_Cell.genes**	**157**
**Muraro_duct.genes**	**27693023**	**Muraro_MJ_et_al_2016_Duct_Cell.genes**	**1280**
**Muraro_Acinar.genes**	**27693023**	**Muraro_MJ_et_al_2016_Acinar_Cell.genes**	**733**
**Muraro_mesenchyme.genes**	**27693023**	**Muraro_MJ_et_al_2016_Mesenchyme_Cell.genes**	**684**
**Muraro_endothelial.genes**	**27693023**	**Muraro_MJ_et_al_2016_Endothelial_Cell.genes**	**362**
**Bramswig_Alpha_Strong.genes**	**23434589**	**Bramswig_NC_et_al_2013_Alpha_Cell_Strong.gene**	**465**
**Bramswig_Beta_strong.genes**	**23434589**	**Bramswig_NC_et_al_2013_Beta_Cell_Strong.gene**	**451**
**Bramswig_Exocrine_Strong.genes**	**23434589**	**Bramswig_NC_et_al_2013_Exocrine_Cell_Strong.gene**	**279**

Supplementary Table 2: Pancreatic Endocrine and Exocrine Gene Set from three Meta dataset. Published literature from Single cell RNA sequencing and FACs sorted total RNAseq data has been used for create PEEGset. In this study, we used following paper's data:

PMID:27364731 - Single cell transcriptomics of the human endocrine pancreas

PMID: 27693023 - A Single-Cell Transcriptome Atlas of the Human Pancreas

PMID: 23434589- Epigenomic plasticity enables human pancreatic α to β cell reprogramming

Supplementary Table 3:

NAME	SIZE	ES	NES	NOM p-val	FDR q-val
BRAMSWIG_ALPHA_STRONG.GENES	446.000	0.674	1.620	<0.001	0.025
MURARO_ALPHA.GENES	555.000	0.587	1.583	0.009	0.023
WANG_ADULT_ALPHA.GENES	210.000	0.693	1.573	0.004	0.021
MURARO_ACINAR.GENES	727.000	0.401	1.134	0.291	0.646
MURARO_DUCT.GENES	1269.000	0.374	0.998	0.464	0.860
MURARO_DELTA.GENES	243.000	0.364	0.984	0.468	0.755
MURARO_PP.GENES	155.000	0.346	0.924	0.588	0.789
BRAMSWIG_EXOCRINE_STRONG.GENES	277.000	0.341	0.802	0.749	0.986
MURARO_BETA.GENES	930.000	0.274	0.738	0.833	1.000
MURARO_ENDOTHELIAL.GENES	361.000	0.317	0.662	0.845	1.000
MURARO_MESENCHYME.GENES	676.000	0.264	0.598	0.935	0.985

Supplementary Table 3: GSEA enrichment scores for all PEEG Set under study. All alpha cell gene set from three different Meta data are significant in A-D-M mutants and highlighted as red.

Supplementary Table 4

A)

Gene name	53_alpha	68_alpha	70_alpha	53_beta	68_beta	70_beta	P-val
HNF1A **expression**	**1.429458333**	**1.458808333**	**1.58975**	**0.981316667**	**1.18395**	**0.995770833**	**0.007123818**

B)

Motif TFs	p-value	FDR q-value
**HNF1_01**	**7.33E-06**	**7.64E-05**
**RGTTAMWNATT_HNF1_01**	**2.80E-14**	**1.91E-12**

Supplementary Table 4: Bramswig et al., expression for HNF1A in normal alpha and beta cells and motif TFs analysis on alpha specific genes. Table a) shows *HNF1A* gene expression in three alpha and three beta FACs sorted cells. *HNF1A* gsene expression is significant (pval< 0.008) in alpha cells. Bramswig et al., 465 strong alpha specific genes were used to find motif TFs enriched for these genes using online version of GSEA (C3 TFs motif database). Table b) shows two HNF1 TFs motifs are significant for alpha 465 genes.
