# Supplementary material for: ATRX, DAXX or MEN1 mutant pancreatic neuroendocrine tumors are a distinct alpha-cell signature subgroup
Source: Nat Commun. 2018 Oct 12;9:4158. doi: 10.1038/s41467-018-06498-2 (PMC6185985; doi:10.1038/s41467-018-06498-2)
Supplement: Supplementary file 3 — Description of Additional Supplementary Files [file 41467_2018_6498_MOESM3_ESM.pdf]

Description of Additional Supplementary Files

Supplementary Data 1

Description: PanNETs clinical information

Supplementary Data 2

Description: PanNETs Mutational Profile for all samples

Supplementary Data 3

Description: PanNETs tumor purity using ESTIMATE

Supplementary Data 4

Description: Gene list for each Pancreases Endo and Exocrine Gene Set (PEEGset)

Supplementary Data 5

Description: A-D-M mutant PanNETs signature validation on two independent PanNETs dataset

Supplementary Data 6

Description: DEseq2 Differentially Expressed Genes, KEGG Pathways and Motif TFs

Supplementary Data 7

Description: Differentially Methylated Probes and PDX1 significant probes **Supplementary**

Supplementary Data 8

Description: RNAseq log2TPM expression matrix for 33 PanNETs samples
